# Ferrimagnetic Heusler tunnel junctions with fast spin-transfer torque switching enabled by low magnetization

**DOI:** 10.1038/s41565-024-01827-7

**Published:** 2025-01-03

**Authors:** Chirag Garg, Panagiotis Ch. Filippou, Ikhtiar, Yari Ferrante, See-Hun Yang, Brian Hughes, Charles T. Rettner, Timothy Phung, Sergey Faleev, Teya Topuria, Mahesh G. Samant, Jaewoo Jeong, Stuart S. P. Parkin

**Affiliations:** 1 IBM Research—Almaden, San Jose, CA USA; 2 Samsung Semiconductor Inc., San Jose, CA USA; 3 Max Planck Institute for Microstructure Physics, Halle (Saale), Germany

**Keywords:** Magnetic devices, Electronic devices

## Abstract

Magnetic random-access memory that uses magnetic tunnel junction memory cells is a high-performance, non-volatile memory technology that goes beyond traditional charge-based memories. Today, its speed is limited by the high magnetization of the memory storage layer. Here we prepare magnetic tunnel junction memory devices with a low magnetization ferrimagnetic Heusler alloy Mn_3_Ge as the memory storage layer on technologically relevant amorphous substrates using a combination of a nitride seed layer and a chemical templating layer. We switch the magnetic state of the storage layer with nanosecond long write pulses at a reliable write error rate of 10^−7^ and detect a tunnelling magnetoresistance of 87% at ambient temperature. These results provide a strategy towards lower write switching currents using ferrimagnetic Heusler materials and, therefore, to the scaling of high-performance magnetic random-access memories beyond those nodes possible with ferromagnetic memory layers.

## Abstract (web summary)

Owing to its very low magnetization, small currents suffice to write a ferrimagnetic Heusler memory layer in complementary metal–oxide–semiconductor-compatible magnetic tunnel via spin-transfer torque, which can help in shrinking the memory cell and extending the application space for magnetic random-access memory.

## Main

Recently, magnetic random-access memory (MRAM), a non-volatile digital memory that utilizes magnetic tunnel junctions (MTJs) as the memory elements, was introduced as a foundry offering at the 28 nm node utilizing 100 ns write pulses^1^. The MTJ is formed from CoFeB ferromagnetic electrodes that are separated by an ultra-thin crystalline MgO(100) tunnel barrier and exhibit very large tunnelling magnetoresistance at ambient temperature^2,3^. The CoFeB is sufficiently thin that the electrodes exhibit perpendicular magnetization. One of the CoFeB layers acts as the memory layer and is written to ‘0’ and ‘1’ states (that is, opposite magnetizations) by the phenomenon of spin-transfer torque (STT) when a large enough current is passed through the junction^4–7^. The speed, however, is limited by the use of the relatively high magnetization of CoFeB owing to spin-transfer angular momentum conservation^8–10^. Reducing substantially the magnetization of the memory layer would enable lower switching currents at higher speeds as well as a smaller access transistor, thereby enabling higher densities.

Several long-standing challenges have prevented the adoption of low magnetization materials. While many ferromagnetic materials exhibit low magnetization, most of them have low Curie temperatures (<400 K)^11,12^. One approach to lowering the magnetization is the use of ferrimagnetic materials^13,14^. Perhaps the best known ferrimagnetic materials are those composed of rare-earth elements (Gd, Tb) and transition metals (Co, Fe). However, these materials have low Curie temperatures and, moreover, a strongly temperature-dependent magnetization owing to the rare-earth magnetic component^15^, making them unsuitable for MRAM applications^16,17^. By contrast, the ferrimagnetic binary Heusler compounds Mn_3_Ga^18^ and Mn_3_Ge^19^ have two antiferromagnetically aligned magnetic sub-lattices, each formed from Mn^20^, a transition metal, that have a weakly temperature-dependent magnetization. Moreover, these compounds are tetragonal and, therefore, exhibit a strong volume derived perpendicular magnetic anisotropy. These properties make tetragonal ferrimagnetic Heusler alloys ideal candidates for high-speed magnetic memory applications. The challenges with these compounds are their growth as ultra-thin layers, with bulk-like properties, on technologically relevant substrates. Another challenge is to incorporate them as memory layers in MTJs that exhibit high TMR at a low resistance-area product (RA) suitable for STT switching^21^. In such ferrimagnets, the spin polarization of the tunnelling current may be compromised owing to the compensating nature of the two antiferromagnetically coupled Mn sub-lattices^22^.

Here we address these challenges and demonstrate STT-switchable MTJs with a memory layer formed from the tetragonal Heusler ferrimagnet Mn_3_Ge^23–26^. We demonstrate that these MTJs, which exhibit high TMR at low RA values ~10 Ωµm^2^, have much lower switching current densities (~10 MA cm^−^^2^) at short write pulse lengths of 0.5 ns, than for MTJs formed from conventional ferromagnetic electrodes for otherwise comparable thermal stabilities^27,28^. Moreover, these Mn_3_Ge-based MTJs are formed on amorphous SiO_*x*_ and are thermally stable at temperatures above 400 °C, making them compatible with typical complementary metal–oxide–semiconductor (CMOS) back-end-of-line processing.

## Ordered alloy growth on amorphous silicon substrates

A recently developed chemical templating layer (CTL) technique^29,30^ was used to grow the Mn_3_Ge-based MTJ stacks, but here we refine the technique to allow for growth on Si/SiO_*x*_ substrates, which is required for CMOS compatibility. CTLs are binary compounds with the CsCl structure having a (001) texture to promote chemical ordering in single-unit cell thick Heusler alloy films deposited on them, even at ambient temperatures^29^. They also enable the desired perpendicular magnetic anisotropy (PMA) in the Heusler layer^29^. This previous work^29^ used single-crystalline MgO(001) substrates so that such structures are not readily compatible with CMOS technologies. Promoting the growth of the CTL on amorphous SiO_2_ layers is not straightforward. After conducting an extensive exploratory search, we found that binary nitrides with a wide range of lattice constants and having an NaCl structure grow with a (001) texture on amorphous surfaces (Supplementary Table 1). Two of these nitrides, MnN (Supplementary Note 1 and Supplementary Figs. 1–3) and ScN, were selected for developing MTJ stacks. We found that metallic MnN was not as thermally stable as semiconducting ScN at ~400 °C, the annealing temperature required for CMOS back-end-of-line integration.

**Fig. 1:**
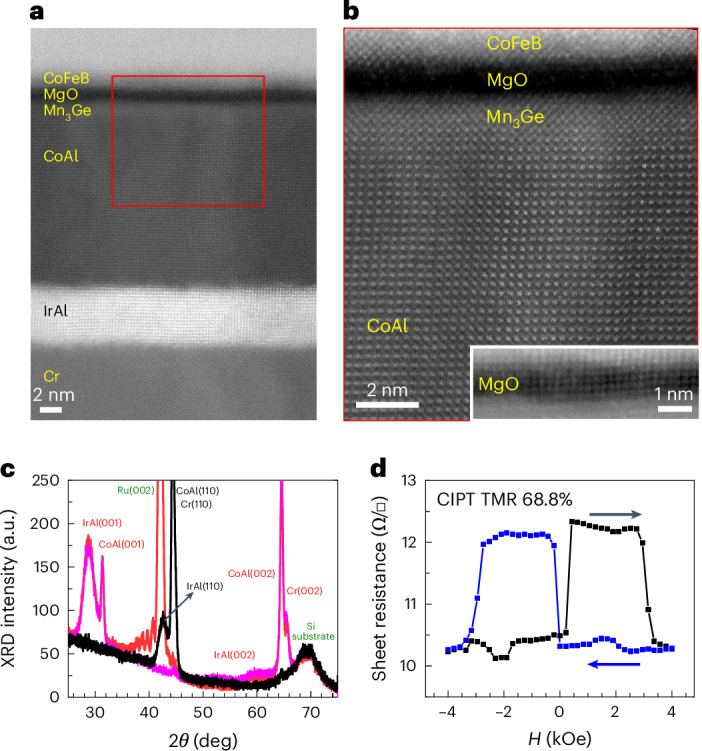
Growth of crystalline CTL on silicon substrates. **a**, HAADF-STEM image of a representative Mn_3_Ge-based MTJ stack grown on a silicon substrate. The stack consists of Si(001)/250 SiO_2_/50 Ta/5 CoFeB/1 ScN/400 Cr/50 IrAl/150 CoAl/19 Mn_3_Ge/14 MgO/14.5 CoFeB/50 Ta/100 Ru. **b**, High-resolution HAADF imaging of the indicated region in **a**, showing the highly epitaxial growth attained even on silicon substrates. Inset: the MgO layer with adjusted dynamic contrast, which shows good crystallinity. **c**, OOP *θ*–2*θ* XRD scans of the stack shown in **a** (in red) and in magenta of the stack: Si(001)/250 SiO_2_/50 Ta/5 CoFeB/1 ScN/400 Cr/50 IrAl/150 CoAl/19 Mn_3_Ge/14 MgO/20 Ta, showcasing the CsCl structure of the CTLs as seen from the (001) and (002) peaks, as indicated. The stack without any nitride layer: Si(001)/250 SiO_2_/50 Ta/5 CoFeB/400 Cr/50 IrAl/150 CoAl/19 Mn_3_Ge/14 MgO/20 Ta (in black) shows (110) peaks of Cr and CoAl and IrAl layers and does not promote Heusler PMA growth. **d**, CIPT *R*–*H* loop showing high TMR from Mn_3_Ge grown stack, similar to the one in **a**, but with a 50 ScN layer instead of a 1 ScN layer. Mn_3_Ge in these stacks is in situ annealed after deposition at 390 °C. All thickness values are given in angstroms.

Cross-sectional high-angle annular dark-field scanning transmission electron microscopy (HAADF-STEM) images, which are presented in Fig. 1a,b, show the epitaxial growth of a Mn_3_Ge layer for an MTJ stack grown on Si/SiO_2_ (stack details in figure caption). The CTL was grown over an ultra-thin ScN seed layer, here just ~1 Å thick. The CTL is formed from a bilayer of IrAl and CoAl, both of which exhibit a CsCl structure with a (001) texture, thereby templating the growth of a chemically ordered Mn_3_Ge (001) layer on top (Fig. 1b). A CTL from a single layer of CoAl also is effective, but we found that higher TMR was possible by using the bilayer CTL. Similarly, ScN can be replaced by other nitrides (Supplementary Table 1). To further demonstrate the role of ScN, in Fig. 1c, we present X-ray diffractograms (XRDs) of the stack with and without any CoFeB reference layer, and with and without the ScN layer (see figure caption for the detailed stack descriptions). Note that a thick 400 Å Cr layer is included within the MTJ stack to allow for current in-plane tunnelling (CIPT) measurements, but the growth of the films is similar without this layer. With the ScN layer, well-defined Cr (002) and both CoAl and IrAl (001) and (002) peaks are observed, whereas without ScN, no such peaks are found, and instead both the CTL and Cr show a (110) texture, which cannot promote PMA in the Mn_3_Ge layer. Thus, the CTL has the desired (001) texture only when grown on Cr/nitride or just nitride underlayers.

## Tunnelling magnetoresistance measurements, DFT predictions and future outlook

MTJs grown in this way show Mn_3_Ge free layers (FLs) with the desired magnetic properties, as seen in the resistance (measured by CIPT) versus out-of-plane (OOP) field (*R*–*H*) hysteresis loop in Fig. 1d. CIPT-TMR measured in this MTJ stack is 69% but when patterned into devices gives rise to higher TMR. The largest TMR that we find for STT-switchable Mn_3_Ge layers is +87% (Supplementary Fig. 4). Note that the TMR is positive, that is, *R*_AP_ > *R*_P_, which is opposite to previously reported results for MTJs with much thicker (300 Å) Mn_3_Ge layers grown without a CTL^22^. Similarly, for thick (50 Å) Mn_3_Ge layers grown with a CTL, we find a negative TMR with a higher value ~−109% (Supplementary Fig. 5). These thick Mn_3_Ge layers are not current switchable owing to their very high magnetic anisotropy. The sign difference in TMR for thin versus thick Mn_3_Ge layers reflects strained versus relaxed Mn_3_Ge layers with the larger in-plane lattice constant and reduced tetragonality of the Mn_3_Ge layer grown with the CTL. Indeed, the TEM images (Fig. 1a,b) show that the thin Mn_3_Ge layer assumes the in-plane lattice parameter (~4.03 Å) of the CoAl CTL.

A positive TMR was found from density functional theory (DFT) calculations (Supplementary Fig. 6) for MTJs that include a Mn_3_Ge layer with the same in-plane lattice constant as that found here for the CoAl CTL. For Mn_3_Ge layers with the bulk lattice constant, previous DFT calculations show a negative TMR^22^. Note that the TMR sign is determined by which of the two Mn–Mn or Mn–Ge ferromagnetic layers have the larger magnetic moment, which, in turn, depends on the in-plane tensile strain according to DFT calculations. Furthermore, the spin polarization of the tunnelling current depends sensitively on this strain so that, surprisingly, the spin polarization from each of the Mn layers can have the same sign and, therefore, higher TMR. Indeed, our DFT calculations (Supplementary Note 2) show the possibility of very high TMR values of up to +400% (Supplementary Fig. 7).

We anticipate that higher TMR values are possible experimentally by further improvements in the structural perfection of the Mn_3_Ge layer itself, as well as the interfaces between this layer and the MgO tunnel barrier. We have found that even the smallest degree of oxidation of the Mn_3_Ge decreases considerably the TMR. This is ameliorated in the radio frequency sputter deposition of the MgO tunnel barrier here by deposition at large offset angles (>~30°) from normal incidence. Further improvements in the degree of chemical ordering of Mn_3_Ge may be accomplished by alternative CTLs or using surfactants. The lattice mismatch between MgO and Mn_3_Ge may be detrimental to TMR and may be improved by dopant engineering of Mn_3_Ge to tune its lattice constant. Another avenue for improvement is via the use of large-scale, multi-chamber dedicated deposition systems, which helps prevent cross-contamination, and cryogenic deposition facilities, the use of both of which often leads to substantial (for CoFeB/MgO-based MTJs) improvements in TMR as compared with laboratory-scale exploratory deposition systems, as used here.

## STT switching, magnetic properties and energy barrier of Mn_3_Ge FL

**Fig. 2:**
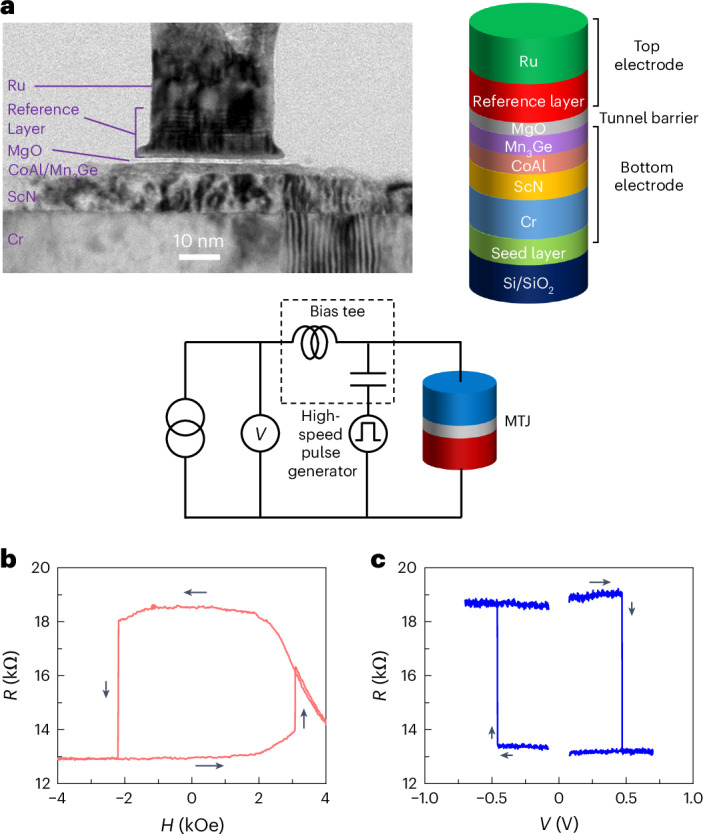
Stack details and switching characteristics of a Mn_3_Ge-FL MTJ device. **a**, Bright-field transmission electron microscopy cross-sectional image of a Mn_3_Ge-FL MTJ device with a diameter of ~36 nm. FL refers to free layer. The MTJ film stack has the following structure: Si(001)/250 SiO_2_/50 Ta/5 CoFeB/1 ScN/400 Cr/100 ScN/10 CoAl/17 Mn_3_Ge/13 MgO/13.5 CoFeB/2.4 Ta/6 Co/10 Pt/(2.5 Co/5 Pt)_*x*3_/7 Co/5.7 Ir/6 Co/5.5 Pt/(5.5 Co/5 Pt)_*x*4_/100 Ru, which is illustrated on the right. The MTJ is connected to a source measurement unit and pulse generator through a bias tee. **b**, *R* versus (OOP) *H* where we see sharp switching of Mn_3_Ge and a gradual rotation of the RL. **c**, *R* versus *V* where the length of the applied voltage pulse is 0.5 ms. *R* is measured with a small bias of 50 mV. Note that a static magnetic field equal to the offset field derived from the *R*–*H* loop is applied for the *R*–*V* measurements to compensate for the fringing dipole field from the top electrode. Mn_3_Ge in this stack is in situ annealed after deposition at 355 °C and all thickness values are in angstroms.

STT measurements were performed on circularly shaped devices that are 30–40 nm in diameter. A typical ~36 nm diameter device is shown in the cross-sectional image presented in Fig. 2a. The Mn_3_Ge forms the FL. The reference layer (RL) is formed from CoFeB that is ferromagnetically exchange-coupled, via a thin Ta spacer layer, to a synthetic antiferromagnetic (SAF) structure composed of two distinct (Co/Pt) multilayers separated by a thin Ir layer^31,32^. The two memory states of the MTJ correspond to the moment of the Mn_3_Ge FL being parallel (P) or antiparallel (AP) to the moment of the lower layer of the RL, with corresponding resistance values, *R*_P_ and *R*_AP_, respectively.

Figure 2b,c shows the switching between the P and AP states of the MTJ, driven by field (*b*) and current (*c*). The MTJ is switched reversibly by using an OOP magnetic field (Fig. 2b), resulting in two well-defined, non-volatile states in the absence of an external magnetic field. By initializing the net moment of the SAF along +*z*, we infer from the magnetic hysteresis loop that the higher resistance belongs to the AP configuration. Therefore, the resulting TMR ($\frac{{R}_{{\rm{AP}}}-{R}_{{\rm{P}}}}{{R}_{{\rm{P}}}}$) is positive, exhibiting a value of +45%. In Fig. 2c, the STT-driven switching of this device is demonstrated by applying voltage pulses of increasing amplitude. Reversible current induced switching back and forth between the P and AP configurations is clearly observed. From the resistance versus voltage (*R*–*V*) hysteresis loop, it can be confirmed that the Mn_3_Ge FL is switching.

**Fig. 3:**
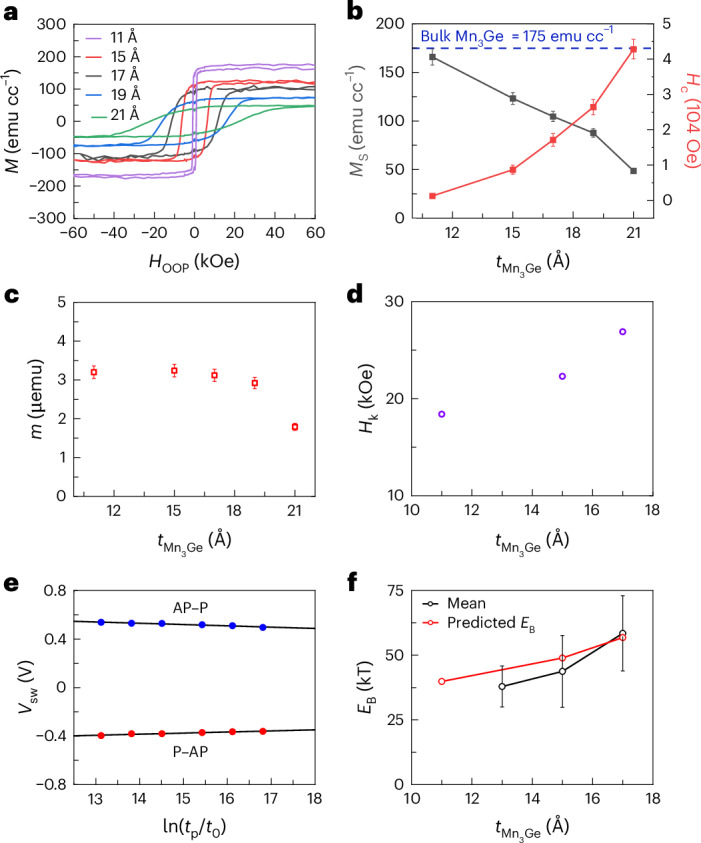
Magnetic measurements and estimation of *E*_B_ for various thicknesses of Mn_3_Ge $({\boldsymbol{t}}_{{\mathbf{Mn}}_{\mathbf{3}}{\mathbf{Ge}}})$. **a**, OOP *M*–*H* curves for the Mn_3_Ge-FL film (Si(001)/250 SiO_2_ /50 Ta/5 CoFeB/1 ScN/400 Cr/100 ScN/10 CoAl/${t}_{{{\rm{Mn}}}_{3}{\rm{Ge}}}$ Mn_3_Ge/12.1 MgO/20 Ta) without any RL. RL refers to reference layer. **b**, Extracted saturation magnetization, *M*_s_ (black), and coercivity, *H*_c_ (red), from *M*–*H* curves. The blue dashed line is a reference for bulk Mn_3_Ge *M*_s_. **c**, Saturation magnetic moment, *m*_s_, normalized to a sample area of 0.2 cm^2^. For *H*_c_, the error bar signifies the field step interval. For *M*_s_ and *m*, the error bar corresponds to a typical standard deviation (s.d.) value of 5% determined from repeated measurements of different pieces of film from the same sample. **d**, *H*_k_ extracted from in-plane *M*–*H* curves (Supplementary Note 3). **e**, Linear fit to the variation of switching voltage (*V*_sw_) with ln$(\frac{{t}_{{\rm{p}}}}{{t}_{0}}$) as per the macrospin model for a device with diameter ~30 nm and ${t}_{{{\rm{Mn}}}_{3}{\rm{Ge}}}=17{\mathring{\rm{A}}}$. *t*_0_ = 1 ns. *V*_sw_ is presented as mean ± s.d. from 20 measurements. s.d. is too small to be visible. **f**, Experimentally obtained *E*_B_ from 35 nm devices (sample sizes of 28 or higher) plotted as mean ± s.d. (black) and theoretically calculated estimate (red) based on *M*_s_ and *H*_k_ from **b** and **d**. The 35 nm group in **f** includes devices in the electrical size range of 32.5–37.5 nm. Mn_3_Ge in this stack is in situ annealed after deposition at 355 °C and all thickness values are in angstroms. s.d. refers to 1 s.d.

The magnetic properties of Mn_3_Ge thin films were studied by growing stacks without the RL. The magnetic OOP hysteresis loops for a series of Mn_3_Ge thin films with varying thickness ${t}_{{{\rm{Mn}}}_{3}{\rm{Ge}}}$ are shown in Fig. 3a. All the films exhibit PMA with high remanence. We find that the saturation magnetization (*M*_s_) and coercivity (*H*_c_) are very sensitive to small changes in ${t}_{{{\rm{Mn}}}_{3}{\rm{Ge}}}$ (Fig. 3b). *H*_c_ more than doubles for every 2 Å increase in thickness reaching a very high value of ~42 kOe for ${t}_{{{\rm{Mn}}}_{3}{\rm{Ge}}}=21{\mathring{\rm{A}}}$. Being able to attain such high values of *H*_c_ can be beneficial for making devices impervious to external fields. The *H*_c_ for thicker films exceeds 70 kOe, the maximum field in our measurement apparatus. For ${t}_{{{\rm{Mn}}}_{3}{\rm{Ge}}}=11{\mathring{\rm{A}}}$, *M*_s_ is ~165 emu cc^−1^, close to the bulk value^26^, but decreases as ${t}_{{{\rm{Mn}}}_{3}{\rm{Ge}}}$ is further increased. This observation likely arises from a subtle shift in the delicate balance of magnetic moments in the Mn–Ge and Mn–Mn layers. The *H*_k_ values were extracted from in-plane magnetic hysteresis loops (Fig. 3d). For ${t}_{{{\rm{Mn}}}_{3}{\rm{Ge}}} > 17{\mathring{\rm{A}}}$, *H*_k_ was too large (>70 kOe) to be measured (Supplementary Note 3).

A useful parameter is the thermal stability factor that is given by $\Delta =\frac{{E}_{\mathrm{B}}}{{kT}}$, where *E*_B_ is the energy barrier to switch the magnetic FL volume (*V*_m_) between the P and AP states and *k* is the Boltzmann constant. For MRAM applications, ∆ should exceed ~50 to meet data retention requirements. For single magnetic domain reversal (macrospin approximation), $\Delta =\frac{{M}_{{\rm{s}}}{V}_{{\rm{m}}}{H}_{{\rm{k}}}}{2{kT}}$, but, typically, smaller values are found, except in small devices (<30 nm)^33,34^. Experimentally, ∆ can be extracted from STT-driven MTJ switching experiments when the switching voltage *V*_sw_ is less than *V*_C0_, the threshold voltage for switching in the absence of thermal fluctuations^35,36^. *V*_sw_ is extracted from *R*–*V* scans (see Fig. 2c for the case of 0.5 ms long pulses). *V*_sw_ is plotted for voltage pulse length (*t*_p_) ranging from 0.5 ms to 100 ms in Fig. 3e for a 30 nm diameter device with ${t}_{{{\rm{Mn}}}_{3}{\rm{Ge}}}=17{\mathring{\rm{A}}}$. Within the macrospin approximation^35–37^, the slope of *V*_sw_ versus $\mathrm{ln}\left(\frac{{t}_{\mathrm{p}}}{{t}_{0}}\right)$ gives $\frac{1}{\Delta }$. Values of ∆ thus obtained for both P → AP and AP → P switching processes are then averaged, and a value of ~60 is estimated for this device. The mean ∆ for devices with a diameter of 35 nm is plotted versus ${t}_{{{\rm{Mn}}}_{3}{\rm{Ge}}}$ in Fig. 3f. These values are in good agreement with estimates based on the values of *M*_s_ and *H*_k_ deduced from Fig. 3b,d. Our results show that Mn_3_Ge FLs may enable scaling the diameter of such MTJs to below ~10 nm.

## Reliable high-speed switching

Having established that Mn_3_Ge FLs have high thermal stability from millisecond STT-switching characteristics, we now discuss their current-driven switching properties at much shorter timescales (≤10 ns), where thermal fluctuations play little role. Instead, the reversal takes place above the threshold current (${I}_{{\rm{C}}0}=\frac{{V}_{\mathrm{C0}}}{{R}_{\mathrm{p}}}$) at which the STT surpasses the intrinsic damping torque^38,39^. In this precessional regime, the switching becomes faster with increasing current. At a given temperature, the reversal starts from a thermally distributed initial state. The switching current *I*_c_ for a given write error rate (WER) is given by the following equation^9,37,39^:

1 $${I}_{\mathrm{C}}=\frac{4e}{{\mu }_{\mathrm{B}}{gP}}\left(\alpha \gamma {E}_{\mathrm{B}}+\frac{{M}_{\mathrm{s}}{V}_{{\rm{m}}}}{4{t}_{\mathrm{p}}}\log \left(\frac{{\pi }^{2}\Delta }{4{\mathrm{WER}}}\right)\right)$$

or alternatively expressed as

2 $${I}_{\mathrm{C}}={I}_{{\rm{C}}0}+{I}_{{\rm{overdrive}}}$$

Here *μ*_B_, *g*, *P*, *α* and *γ* are the Bohr magneton, *g*-factor, spin polarization, Gilbert damping constant and gyromagnetic ratio, respectively. In the long-pulse limit, *I*_C_ reaches a saturation lower bound value that is given by ${I}_{{\rm{C}}0}=\frac{4e\alpha \gamma {E}_{\mathrm{B}}}{{\mu }_{\mathrm{B}}{gP}}$ and which is governed by *E*_B_ and *α*. The efficiency of the switching process is often characterized by the term $\frac{\Delta }{{I}_{{\rm{C}}0}}$, which should be as high as possible. $\frac{\Delta }{{I}_{\mathrm{C0}}}$ can be increased, for example, by suppression of spin pumping by using an MgO dielectric cap layer on top of the FL^40,41^ or making use of two tunnel barriers^42^ to increase the spin torque. In the short-pulse limit, the switching current is increased above the saturation lower bound value by the term ${I}_{{\rm{overdrive}}}=\frac{4e}{{\mu }_{\mathrm{B}}{gP}}\frac{{M}_{\mathrm{s}}{V}_{{\rm{m}}}}{4{t}_{\mathrm{p}}}\log \left(\frac{{\pi }^{2}\Delta }{4{\mathrm{WER}}}\right).$ This term scales with ${t}_{\mathrm{p}}^{-1}$ and the magnetic moment of the FL.

It is clear from equation (1) that a high *M*_s_ is detrimental for current-driven switching at short pulses, although, for typical ferromagnets, it contributes towards a high *E*_B_. By contrast, ferrimagnets that have a low *M*_s_ and high *H*_k_, such as Mn_3_Ge used here, can attain a high *E*_B_ while reducing *I*_overdrive_. We now consider the overdrive term by studying the ratio $\frac{{J}_{\mathrm{C}}}{{J}_{{\mathrm{C0}}}}$, where *J*_C_ is the switching current density and *J*_C0_ is the threshold saturation lower bound current density. For a given *t*_p_, equation (1) can be rewritten as

3 $$\frac{{J}_{\mathrm{C}}}{{J}_{{\rm{C}}0}}=\left[1+\frac{{\tau }_{\mathrm{D}}}{{t}_{\mathrm{p}}}\left(\frac{1}{2}\log \left(\frac{{\pi }^{2}\Delta }{4{\mathrm{WER}}}\right)\right)\right]$$

**Fig. 4:**
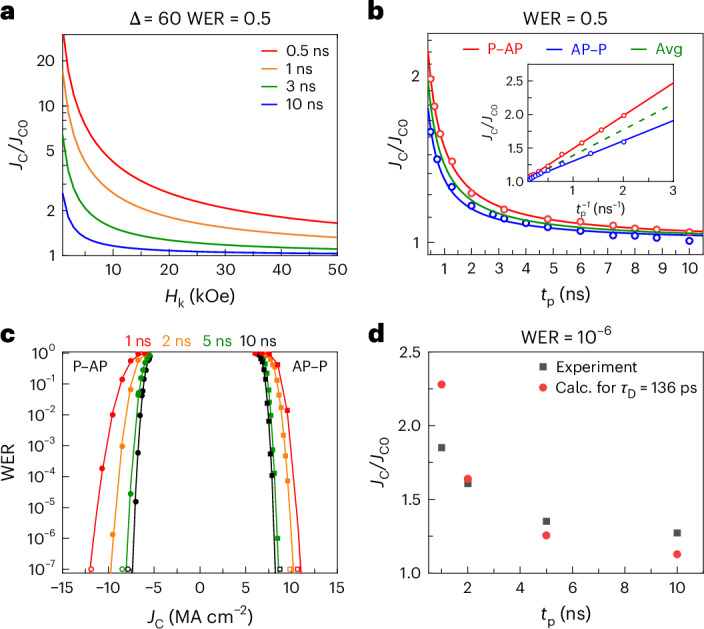
Scaling of switching currents at high speeds. **a**, $\frac{{J}_{\mathrm{C}}}{{J}_{\mathrm{C0}}}$ calculated for WER = 0.5, ∆ = 60 and *α* = 0.01 using equation (3). **b**, $\frac{{J}_{\mathrm{C}}}{{J}_{\mathrm{C0}}}$ versus *t*_p_ for Mn_3_Ge-FL MTJ with ${t}_{{{\rm{Mn}}}_{3}{\rm{Ge}}}=17{\mathring{\rm{A}}}$ (same device from Fig. 3e). Measured data for P–AP (red) and AP–P (blue) and the average (green) are shown using fits to equation (3). The linear scaling of $\frac{{J}_{\mathrm{C}}}{{J}_{\mathrm{C0}}}$ with ${t}_{\mathrm{p}}^{-1}$ is shown in the inset. The *J*_C0_ for the P–AP, AP–P and ‘Avg’ correspond to 5.38 MA cm^−^^2^, 6.71 MA cm^−^^2^ and 6.05 MA cm^−^^2^. **c**, Measured *J*_C_ versus WER for different *t*_p_ (solid points) with fits to the WER (solid lines) for the same device. The open symbols are measurements where no error was detected for 10^7^ switching events and a WER of 10^−7^ was assumed as the upper bound. **d**, $\frac{{J}_{\mathrm{C}}}{{J}_{\mathrm{C0}}}$ from experiment in **c** (black) and from calculations based on equation (3) (red) are plotted for different *t*_p_.

Here *τ*_D_ is the characteristic timescale of switching, which equals $\frac{1}{\alpha \gamma {H}_{\mathrm{k}}}$. Calculated values of $\frac{{J}_{\mathrm{C}}}{{J}_{\mathrm{C0}}}$ are plotted in Fig. 4a as a function of *H*_k_ using equation (3). Note that as *H*_k_ is varied, *M*_s_ is correspondingly adjusted to maintain the value of ∆ to be 60. The value of *α* = 0.01 was chosen as it is close to the experimentally obtained *α* for magnetic FLs composed of Mn_3_Ge^43^ and CoFeB^41^. For longer pulse lengths (~10 ns), $\frac{{J}_{\mathrm{C}}}{{J}_{{\rm{C}}0}}$ is small and relatively insensitive to *H*_k_. As clearly shown in Fig. 4a, for low *H*_k_ and short *t*_p_, *J*_C_ is calculated to increase to more than an order of magnitude higher than *J*_C0_, but, on the other hand, ferrimagnetic layers with a low *M*_s_ and high *H*_k_ will dramatically reduce $\frac{{J}_{\mathrm{C}}}{{J}_{{\rm{C}}0}}$ even at sub-nanosecond speeds.

As shown in equation (3), there is a finite probability (WER) that a device will not switch with the writing pulse that is used to characterize the reliability of the writing process of an MTJ. Results for the Mn_3_Ge-FL device showcased earlier in Fig. 3e are shown in Fig. 4b,c. Figure 4b shows how $\frac{{J}_{\mathrm{C}}}{{J}_{{\rm{C}}0}}$ at a higher WER of 0.5 varies for *t*_p_ ranging from 0.5 ns to 10 ns. Experimental data for both AP–P and P–AP switching processes are fitted with equation (3) to obtain *J*_C0_ (6.05 MA cm^−^^2^) and *τ*_D_ (136 ps). On the basis of the fitted data, the STT switching efficiency $\frac{\Delta }{{I}_{{\rm{c}}0}}$ is ~1.37. This value is comparable to those reported in the literature for conventional ferromagnetic materials^7,44^. For 1 ns long current pulses, we observe the increase in *J*_C_ over *J*_C0_ to be ~38% (for WER = 0.5) in our device. For MTJs with CoFeB FLs, this increase is typically more than 200% (refs. ^28,45^). This is due to the large difference in *M*_s_ for these two materials.

During device operations, a much lower WER is required so *J*_C_ must be increased. *J*_C_ at WER as low as 10^−7^ are plotted for several values of *t*_p_ in Fig. 4c. Fits to the WER versus *J*_C_ plots for each *t*_p_ are shown as solid lines (Methods). For some *J*_C_, no errors were detected for the maximum number of switching attempts (10^7^) that we performed in our study so they are plotted at WER = 10^−7^ (hollow points). From these data, a plot of $\frac{{J}_{\mathrm{C}}}{{J}_{\mathrm{C0}}}$ versus *t*_p_ for WER = 10^−6^ is shown in Fig. 4d. The values of $\frac{{J}_{\mathrm{C}}}{{J}_{\mathrm{C0}}}$ agree well with those calculated using the value of *τ*_D_ obtained from the data in Fig. 4b. In particular, $\frac{{J}_{\mathrm{C}}}{{J}_{\mathrm{C0}}}$ for 1 ns long current pulses at WER = 10^−6^ is ~1.85, which is again considerably smaller than that for conventional ferromagnetic FLs^28^. This shows the suitability of Mn_3_Ge FL for achieving highly reliable switching at short pulse lengths without a notable increase in *J*_C_.

## Conclusion

In summary, we have developed CMOS-compatible MTJ non-volatile memory elements that use small switching current densities (~10 MA cm^−^^2^) at a short write pulse length of 0.5 ns. This is enabled by the use of a ferrimagnetic Heusler memory layer that has a very low saturation magnetization, about six times lower compared with the ferromagnetic CoFeB-based FLs used in today’s MRAM products. The low switching currents that we have demonstrated will allow for the use of minimum size drive transistors, thereby enabling a reduction in the footprint of the memory cell and overcoming a roadblock to the widespread application of spintronic technologies based on MTJ devices. The family of Heusler compounds is so extensive that one can anticipate that other members of this family will have superior performance to that presented here.

## Methods

### Magnetic measurements

The magnetic measurements of the Mn_3_Ge film were conducted using a SQUID-VSM magnetometer (Quantum Design).

### Device sizes

The film stacks were patterned into circularly shaped MTJ devices, 30–40 nm in diameter, using electron-beam lithography and Ar ion-beam milling. The physical size of the MTJs, as determined through TEM cross-sectional images, can slightly vary from the nominal size. We find that the electrical size estimated using *R*_P_ and the value of resistance-area (RA) product obtained from CIPT measurements agrees well with the size determined from TEM. Therefore, we use the electrical size to refer to the device size throughout the paper.

### Film growth, stack and characterization

The samples used in these studies were prepared using an ultra-high vacuum chamber with a base pressure of ~10^−9^ Torr. While the Ta and Mn_3_Ge layers were deposited using ion-beam deposition (with Kr gas) at a pressure 10^−4^ Torr, all the other layers were deposited by d.c. magnetron sputtering at an Ar sputter gas pressure of 3 mTorr (the MgO tunnel barrier was deposited by radio frequency sputtering at the same pressure). The nitride layers were deposited by reactive sputtering from a metallic target with a gas mixture of Ar and N_2_. The ScN layer was deposited from a Sc target with sputter gas consisting of 85% Ar and 15% N_2_. The ScN thickness can range anywhere from an interfacial layer (nominally 1 Å) up to several 100 Ås. All the films were deposited at room temperature. All MTJ stacks utilizing a CoFeB RL, in order to set PMA in the CoFeB (either by itself or in SAF structure), were in situ annealed at 300 °C after the Ta layer was deposited. Small changes in the reported annealing temperatures are related to differences in structure that may require small changes in optimal deposition and annealing conditions. As mentioned in the main text, a thick 400 Å Cr layer is included within MTJ stack to allow for CIPT measurements. The SAF structure consisted of an upper (Co/Pt) multilayer designed to have a higher moment than the lower CoFeB/Ta/(Co/Pt) multilayer of the film structure. The XRDs in Fig. 1c are normalized to the Si substrate peak. We determined the film stoichiometry by Rutherford backscattering measurements on calibration films.

### WER measurements

WER measurements were performed by connecting a Keithley 2602A multimeter and a pulse generator through a bias tee to the MTJ device. The 2602A unit was used for sending the reset pulse to set the initial state of the MTJ and reading the resistance of the MTJ. Either a Picosecond Pulse Generator 10070A or a Tektronix AWG610 was used for sending the write pulse. The 10070A has a rise time of 55 ps and was used for the shorter writing pulses. Both the reset and write resistance states were measured after the state of the MTJ was set. WER was determined by first setting the MTJ to either the AP or P state using an appropriate current pulse and then applying a switching current pulse. The state of the MTJ was then read with a much smaller current sensing pulse. *J*_C_ for WER = 0.5 is obtained after accumulating the WER for different *J*_C_ and using that data to obtain the fit for *J*_C_ at WER = 0.5 (Fig. 4b). For deeper error rate measurements (from Fig. 4c), measurement at a particular *J*_C_ is run until any of these conditions are satisfied; either 10 errors are accumulated or 10^7^ trials have been performed. The WER then obtained is passed through the inverse of the standard normal cumulative distributive function and then linearly fitted against *J*_C_ (solid lines) in Fig. 4c. The smallest WER that we can measure is limited by the amount of time that it takes for a measurement to conclude. WER measurements up to 10^−6^ or 10^−7^ can be used to estimate *J*_C_ at lower WER.

## Online content

Any methods, additional references, Nature Portfolio reporting summaries, source data, extended data, supplementary information, acknowledgements, peer review information; details of author contributions and competing interests; and statements of data and code availability are available at 10.1038/s41565-024-01827-7.

## Supplementary information

Supplementary Information Supplementary Table 1, Figs. 1–13 and Notes 1–5.

## Data Availability

The data that support the findings of this study are available from the corresponding authors upon reasonable request.
