# Supplementary material for: Ferrimagnetic Heusler tunnel junctions with fast spin-transfer torque switching enabled by low magnetization
Source: Nat Nanotechnol. 2025 Jan 3;20(3):360–5. doi: 10.1038/s41565-024-01827-7 (PMC11919757; doi:10.1038/s41565-024-01827-7)
Supplement: Supplementary file 1 — Supplementary Table 1, Figs. 1–13 and Notes 1–5. [file 41565_2024_1827_MOESM1_ESM.pdf]

nature nanotechnology

Supplementary information https://doi.org/10.1038/s41565-024-01827-7

Ferrimagnetic Heusler tunnel junctions with fast spin-transfer torque switching enabled by low magnetization

In the format provided by the authors and unedited

Table of Contents

Supplementary Table 1	Exploration of different nitrides as the seed layer for the growth of Mn_3Ge free layer magnetic tunnel junction
Supplementary Note 1	Selection and growth of suitable nitrides
Supplementary Figure 1	Growth of crystalline chemical templating layer on amorphous substrates.
Supplementary Figure 2	The high CoAl $I(001)/I(002)$ peak intensity ratios, as extracted from Supplementary Figure 1.
Supplementary Figure 3	FWHM data obtained from the Mn_xN XRD peak of Supplementary Figure 1, along the gamma direction.
Supplementary Figure 4	Resistance versus field (*R-H*), magnetic hysteresis loop for a 35 nm MTJ device with Mn_3Ge free layer, showing a TMR of 87%.
Supplementary Note 2	Density functional theory calculations of Mn_3Ge
Supplementary Figure 5	High TMR from MTJ stack with Mn_3Ge FL of 50 Å.
Supplementary Figure 6	The density of states of Mn_3Ge with in-plane lattice constant a = 4.03 Å calculated by LDA and QSGW methods.
Supplementary Figure 7	TMR calculated for Mn_3Ge/MgO/Fe MTJ with in-plane lattice constant a = 4.03 Å
Supplementary Note 3	Extraction of anisotropy field magnetic properties of Mn_3Ge thin film
Supplementary Figure 8	Normalized magnetization vs H curves supporting the extraction procedure in Supplementary Note 3.
Supplementary Figure 9	Coefficient of variability for TMR and J_C
Supplementary Figure 10	Nominal vs electrical size comparison of MTJ devices.
Supplementary Figure 11	Biased dependence of TMR for a representative device from MTJ stack with t_{Mn_3Ge} = 17 Å.
Supplementary Note 5	Size dependence of device E_B
Supplementary Figure 12	Figure supporting the discussion in Supplementary Note 5.
Supplementary Figure 13	Typical CIPT measurement results for one of the MTJ stacks.

Supplementary Table 1: Exploration of different nitrides as the seed layer for the growth of Mn_3Ge free layer magnetic tunnel junction

**Nitride**	**TMR (%)**	**RA ($\Omega\cdot\mu m^2$)**
ScN	56.6	5.58
TiN	60.8	7.75
VN	58.8	7.13
CrN	51.9	4.88
MnN	62.3	19.4
TaN	56.9	9.11

Table shows current in-plane tunneling derived tunneling magnetoresistance (CIPT-TMR) values for Mn_3Ge layer stacks grown on Si substrates utilizing the nitride/CTL (chemical templating layer) concept. These nitrides may not be stoichiometric. All film stacks are comprised of: 50 Ta/ 5 CoFeB/ Nitride / 400 Cr/ 50 IrAl/ 150 CoAl/ 13-19 Mn_3Ge/ 14-17 MgO/ 13-14.5 CoFeB/ 50 Ta/ 100 Ru, all thicknesses are in Å. Mn_3Ge is annealed after deposition at ~390°C and there is a second annealing step at ~300°C after all layers are deposited to set the perpendicular magnetic anisotropy (PMA) in the CoFeB layer. The nitride thicknesses range from 1-10 Å, except for MnN which is 300Å thick.

Supplementary Note 1: Selection and growth of suitable nitrides

We show that nitrides extend the CTL technique of ordered growth of Heusler films from MgO (100) single crystal substrates to amorphous SiO_x. Here we discuss in detail the case of a metallic Mn_xN layer (other nitrides are also similar). Mn_xN films with different chemical compositions, varied from MnN to $Mn_{4.8}N$, were prepared by reactive magnetron sputtering using varying mixtures of Ar – N_2 sputter gas. This allows the lattice constant of the Mn_xN layer to closely match that of the CTL.

We illustrate the growth of ultra-thin, 10 Å thick Heusler layers formed from Mn_3Sb, using a combination of Mn_xN and CoAl CTL underlayers in Supplementary Figure 1. The detailed structure is as follows: Si(001)/ 250 SiO_2/ 50 Ta/ 3 CoFeB/ 300 Mn_xN/ 300 CoAl/ Mn_3Sb or Mn_3Ge / 20 MgO/ 20 Ta, all thicknesses are in Å. The variation of the lattice constant of the

Mn_xN layer as a function of nitrogen content is shown in Supplementary Figure 1a and b. *θ-2θ* x-ray diffraction (XRD) scans in Supplementary Figure 1a show that the (002) Mn_xN peak shifts to lower *2θ* angles with increasing nitrogen content. Thus, the Mn_xN out-of-plane lattice parameter can be varied considerably from ~3.76 to ~4.26 Å with increasing nitrogen content, as summarized in Supplementary Figure 1b. When the nitrogen content is ~ 2.5 the lattice constant of the Mn_xN layer matches closely with that of the CoAl CTL. However, we find that well-ordered CoAl can be prepared for a wide range of nitrogen content within the Mn_xN layer as seen in Supplementary Figure 2. Supplementary Figure 3 shows the CoAl peak intensity ratio of the ordering for the different MnN seed layers. The chemically ordering within the CoAl layer gives rise to the (001) peak shown in Supplementary Figure 1a. As can be seen in the figure, a strong (001) peak, that varies little in *2θ* is observed for a wide range of x between ~2 and ~4. For the same range of nitrogen content, the surface of the film stacks was found to be very smooth. As can be seen in Supplementary Figure 1c, the root-mean-square roughness (R_{rms}) of the surface topography, found from atomic force microscopy (AFM) studies, was less than 3Å for x between ~2 and ~4.

Using this combination of Mn_xN and CTL underlayers very thin (~1 nm) Heusler films with excellent magnetic properties were obtained. Exemplary Magneto-optical Kerr effect (MOKE) perpendicular magnetic hysteresis loops are shown in the inset to Supplementary Figure 1d for 10 Å-thick Mn_3Sb and 8 Å-thick Mn_3Ge films. Both the Mn_3Sb and Mn_3Ge layers were deposited at room temperature (RT) but the Mn_3Ge layer was in-situ annealed at ~ 340 ^oC in ultra-high vacuum for 30 min before the capping layers were deposited. These both show excellent PMA with square hysteresis loops. This shows that the Heusler layers are highly thermally stable. The dependence of film coercivity (H_c) on the thickness of the Mn_3Ge layer (t_{Mn_3Ge}) is shown in Supplementary Figure 1d.

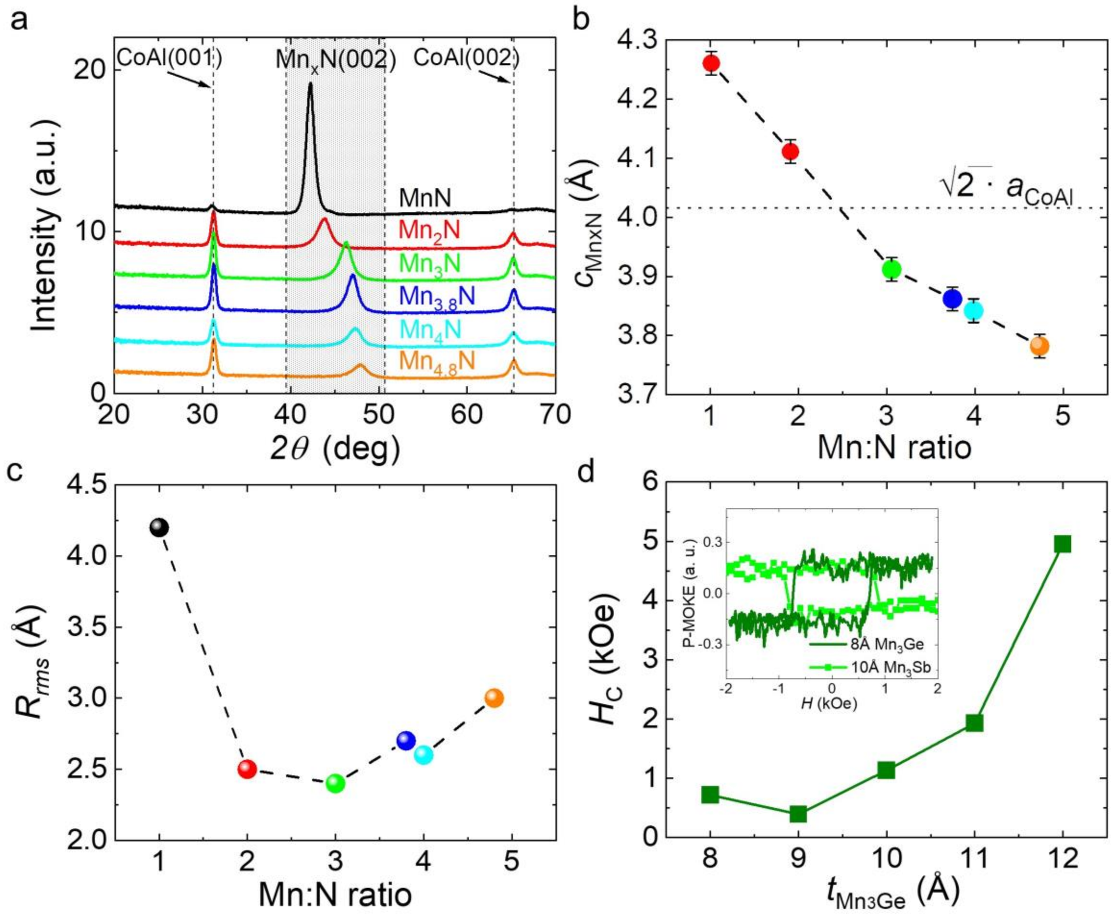

Supplementary Figure 1.

Growth of crystalline chemical templating layer on amorphous substrates. **a,** Out-of-plane θ-2θ XRD scans of CoAl films grown on Mn_xN with varying compositions. The dotted lines indicate the positions of the (001) and (002) CsCl-CoAl peaks. **b,** Dependence of the Mn_xN out-of-plane lattice constant on the Mn:N ratio extracted from (a). Error bars correspond to step size of the scan. The dotted line indicates the value of the CoAl lattice constant after a 45° in-plane rotation. **c,** Surface roughness of the films shown in (a) as measured by atomic force microscopy. **d,** H_c dependence of Mn_3Ge films with thickness t_{Mn3Ge} grown on top of the optimized CoAl layer. Inset shows strong PMA in the magnetic hysteresis loops of 8 Å Mn_3Ge and 10 Å Mn_3Sb films measured using p-MOKE. The stack structure used for a-d is: Si(001)/ 250 SiO_2/ 50 Ta/ 3 CoFeB/ 200 Mn_3N/ 300 CoAl/ [Mn_3Ge or Mn_3Sb]/ 20 MgO/ 20 Ta, all thicknesses are in Å.

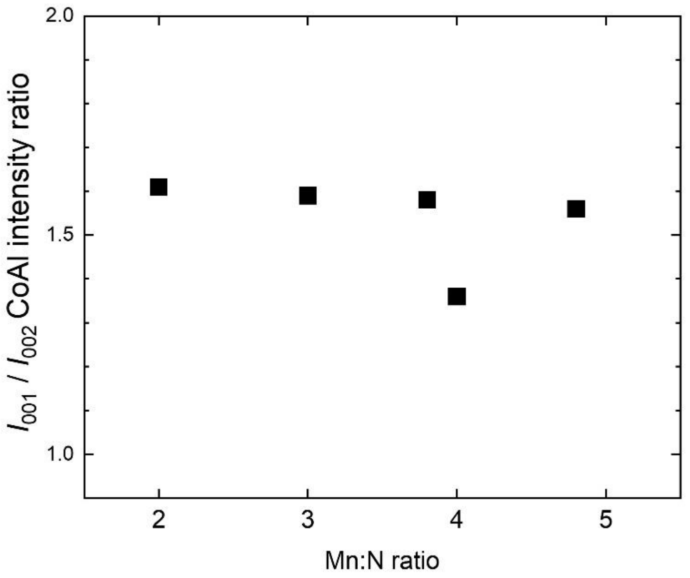

Supplementary Figure 2.
The high CoAl $I(001)/I(002)$ peak intensity ratios, as extracted from Supplementary Figure 1, are indicative of the high chemical ordering within these layers. Moreover, this intensity ratio remains almost constant for a sequence of films prepared with different Mn-N rations. For the disordered case this ratio is zero. We note that the ratio takes a value of 1.17 for a single crystal of CoAl as detailed in the Pearson's crystallographic database.

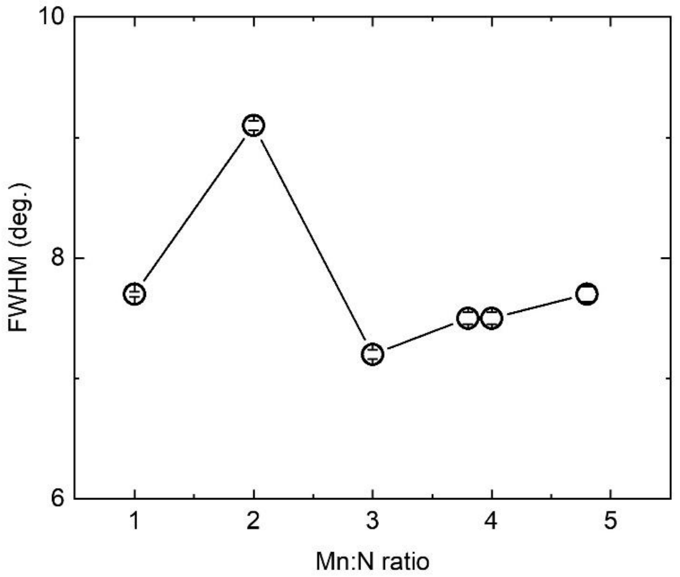

Supplementary Figure 3.

FWHM data obtained from the Mn_xN XRD peak of Supplementary Figure 1, along the gamma direction. We found that the FWHM is smallest when x = 3 in the Mn_xN film. Error bars are derived from the fitting function.

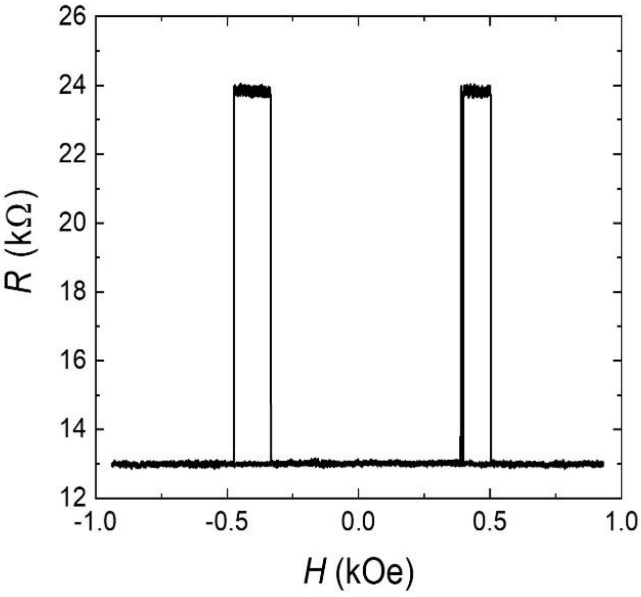

Supplementary Figure 4:

Resistance versus field (*R*-*H*), magnetic hysteresis loop for a 35 nm MTJ device with Mn_3Ge free layer, showing a TMR of 87%. The stack is as follows: 50Ta/ $5CoFeB_{20}$/ $300Mn_xN$/ 400Cr/ 50IrAl/ 150CoAl/ $13Mn_3Ge$/ anneal at ~340°C/ 17MgO/ 13CoFeB/ 50Ta/ 100Ru/ anneal at ~300°C, all thicknesses are in Å.

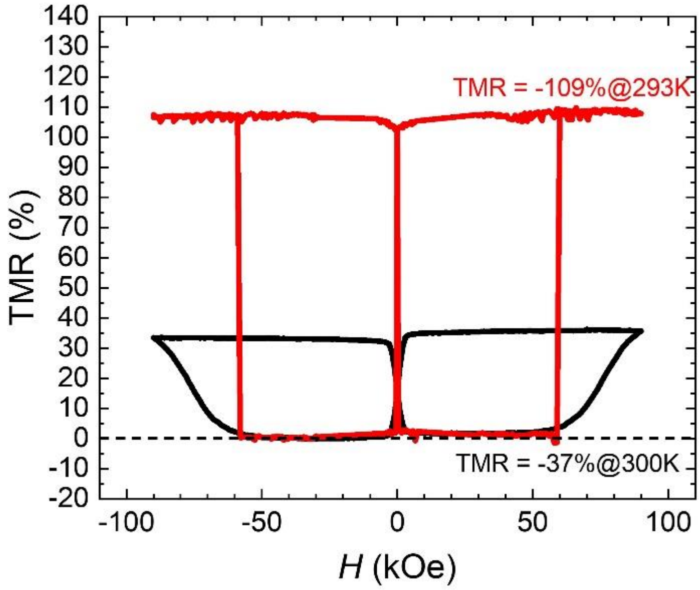

Supplementary Figure 5:
In red, high TMR from Mn_3Ge of 50 Å where Mn_3Ge assumes its bulk properties of Mn-Mn layer moment aligning with the total moment and TMR is negative (measured at 293K). For comparison, in black, previously measured TMR on 300 Å bulk Mn_3Ge [1]. Even the applied 9T in magnitude field is not large enough to fully saturate the 300 Å Mn_3Ge, thus the black curve shows a small shift in resistance due to that. Whereas the red curve of 50 Å Mn_3Ge is well saturated, and resistance follows the fully saturated states.

Supplementary Note 2: Density functional theory calculations of Mn_3Ge

In order to study the electronic structure and transport properties of Mn_3Ge deposited on CoAl substrate we performed density functional theory (DFT) calculations of the Mn_3Ge crystal structure with fixed in-plane lattice constant a = 4.03 Å (which equals to the lattice constant of CoAl) using the VASP program [2] with projector augmented wave (PAW) potentials [3,4] and Perdew-Burke-Ernzerhof (PBE) GGA/DFT functional [5]. We found that for fixed in-plane lattice constant a = 4.03 Å the relaxed out-of-plane lattice constant equal c = 5.964 Å (that corresponds to the dimensionless out-of-plane lattice constant c' = c/(2a) = 0.74). Note that a = 4.03 Å is just 1% smaller than the in-plane lattice constant a_c = 4.06 Å of the cubic phase of Mn_3Ge [6] (that corresponds to the dimensionless out-of-plane lattice constant c' = $c/(2a) = 1/\sqrt{2} \approx 0.707$). The convergence of the results was verified by varying the number of divisions in reciprocal space from 10×10×10 to 18×18×18 and the energy cutoff from 400 to 520 eV.

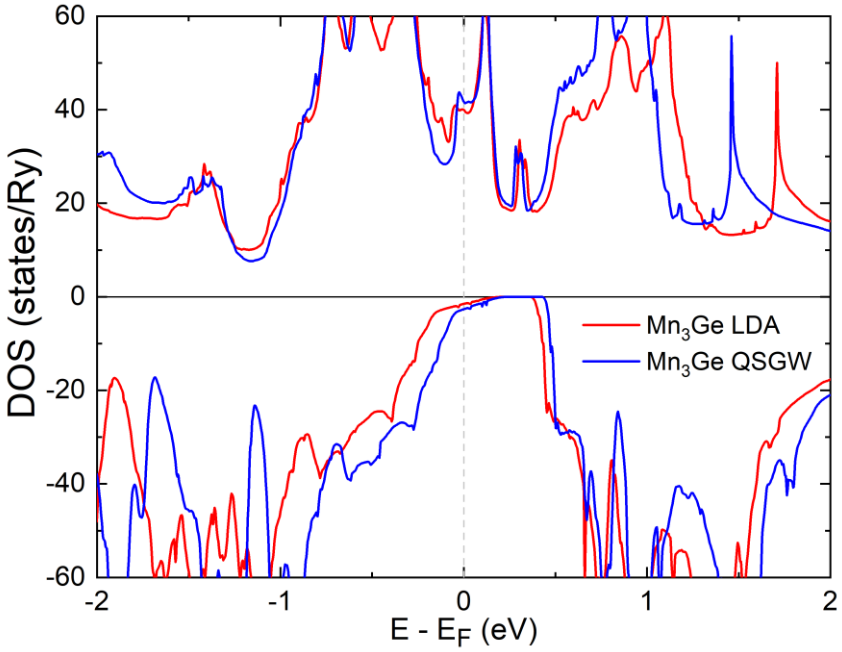

Supplementary Figure 6: The density of states of Mn_3Ge with in-plane lattice constant a = 4.03 Å calculated by LDA and QSGW methods.

The electronic structure of Mn_3Ge with in-plane lattice constant a = 4.03 Å and out-of-plane lattice constants c' = 0.74 was calculated using the full-potential all-electron linear muffin-tin orbital (LMTO) approach [7] with Barth-Hedin LDA/DFT functional [8], and also using the quasiparticle self-consistent GW (QSGW) method that is known to describe band gaps and other properties of materials with moderate *e-e* correlations significantly better than DFT [9–

11]. The density of states (DOS) calculated by LDA and QSGW methods is presented Supplementary Figure 6. One can see that in both approaches the minority DOS has a valley near the Fermi energy resulting in large spin polarization (SP) of Mn_3Ge for a = 4.03 Å. In particular, the spin polarization obtained by LDA is SP_{LDA} = 0.92, and spin polarization obtained by more accurate QSGW method is SP_{QSGW} = 0.88. The magnetic moment was found to be 1.02 μ_B in LDA and 1.03 μ_B in QSGW, that is close to the magnetic moment of Mn_3Ge in cubic phase m_c = 1.00 μ_B [6].

The transport properties of Mn_3Ge/MgO/Fe magnetic transport junction (MTJ) device with fixed in-plane lattice constant a = 4.03 Å were calculated using a tight-binding linear muffin-tin orbital method in the atomic sphere approximation (TB-LMTO-ASA) with the local density approximation of DFT for the exchange-correlation energy [12,13]. Relaxed positions of atoms at the Mn_3Ge/MgO interfaces (for both, the Mn-Mn and Mn-Ge terminations of the interface) were determined using the VASP molecular dynamic program [2]. The O-top configuration was found to be the most stable configuration (as compared with Mg-top and hollow) for both terminations at the Mn_3Ge/MgO interface (in agreement with Ref. [14]). For Fe/MgO interface the atomic positions from Ref. [15] were used.

Even though the Mn_3Ge/MgO interface can be very smooth (see, e.g., Ref. [1]) inevitably there will be atomic scale fluctuations in the morphology of the Mn_3Ge layer that gives rise to regions with Mn–Mn and Mn–Ge terminations, due to the fundamental underlying structure of the Heusler compound. The simplest way to model such fluctuations is to average the transmission functions over the different terminations separately for parallel (P) and antiparallel (AP) configurations of magnetization of the Mn_3Ge and Fe electrodes in the Mn_3Ge/MgO/Fe MTJ, assuming that the MgO thickness is the same across the device. The tunneling magneto resistance (TMR) is this simple model is calculated as TMR = $(T_P - T_{AP})\ /T_{AP}$, where transmission in the parallel configuration is given by $T_P = [T_{\uparrow\uparrow}(MnMn) + T_{\downarrow\downarrow}(MnMn) + T_{\uparrow\uparrow}(MnGe) + T_{\downarrow\downarrow}(MnGe)]/2$ and transmission in the antiparallel configuration is given by $T_{AP} = [T_{\uparrow\downarrow}(MnMn) + T_{\downarrow\uparrow}(MnMn) + T_{\uparrow\downarrow}(MnGe) + T_{\downarrow\uparrow}(MnGe)]/2$. (Here two arrows denote direction of the magnetization of Mn_3Ge and Fe, correspondingly, and MnMn or MnGe denote the termination at the Mn_3Ge/MgO interface). The calculated TMR is shown in Supplementary Figure 7.

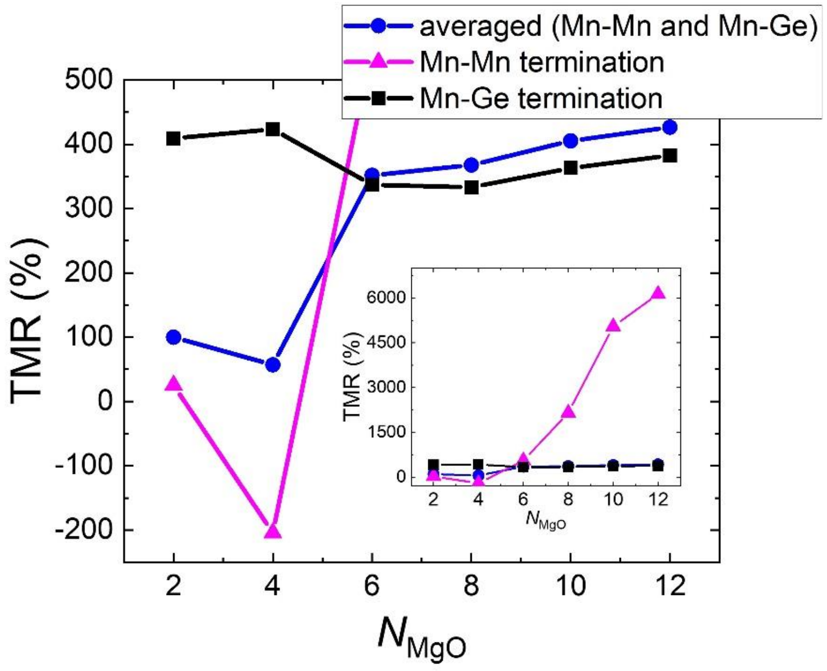

Supplementary Figure 7: TMR calculated for Mn_3Ge/MgO/Fe MTJ with in-plane lattice constant a = 4.03 Å for all three cases of Mn-Mn or Mn-Ge termination and with an assumption of equal areas occupied by Mn–Ge and Mn–Mn terminations at the Mn_3Ge/MgO interface shown as a function of the number of MgO layers, N_{MgO}.

One can see that TMR is 100% for N_{MgO} = 2 and 60% for N_{MgO} = 4 and varies from 360% to 430% for N_{MgO} ranging from 6 to 12. The high TMR values (~ 400%) at N_{MgO} >= 6 is a consequence of the high spin polarization of the near-cubic crystal structure of Mn_3Ge at a = 4.03 Å. Lower TMR values (~100%) at N_{MgO} <= 4 is a consequence of the presence of the interface resonance states localized near the Fe/MgO interface at the Fermi energy in Fe minority channel that leads to enhanced AP transmission (and therefore lower TMR) at small values of N_{MgO}.

Supplementary Note 3: Extraction of anisotropy field magnetic properties of Mn_3Ge thin film

The effective anisotropy field (H_k) of the Mn_3Ge film used in our study was estimated by obtaining the area enclosed (light green) between the OOP (black) and IP (blue) measurements of M-H (magnetization vs field). M-H was measured using a Quantum Design VSM-SQUID magnetometer capable of applying maximum field of 7 Tesla. The area enclosed gives the energy density difference between the OOP and IP configuration which when normalized by the saturation magnetization gives us the value of the effective anisotropy field. The Mn_3Ge films used for these measurements have been described earlier in the main text and their stack is: Si(001)/ 250Å SiO_2/ 1ScN/ 10CoAl/ 't_{Mn_3Ge}' Mn_3Ge/ 20 MgO/ 20 Ta, all thicknesses are in Å. For t_{Mn_3Ge} = 11 Å, 15 Å, 17 Å, the normalized m-H data and the enclosed curves illustrating our procedure are shown in Supplementary Figure 8a-c. We are not able to measure the anisotropy field for higher t_{Mn_3Ge} as the field range of our magnet (7T) is not sufficient to completely saturate the magnetization during the IP measurement.

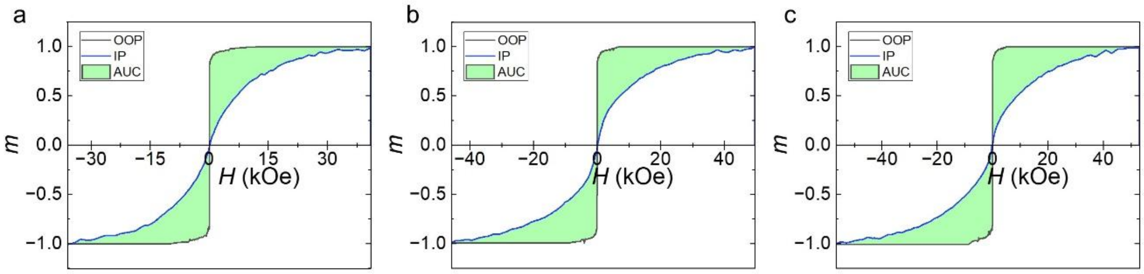

Supplementary Figure 8. Normalized magnetization (m) vs H curves for OOP (black) and IP configurations (blue). The enclosed area is shaded in light-green. (a-c) correspond to t_{Mn_3Ge} = 15 Å b, t_{Mn_3Ge} = 11 Å and c, t_{Mn_3Ge} = 17 Å, respectively.

Supplementary Note 4: Variation in TMR and J_{C10ns} for 35 nm devices.

In order to assess the variability of important parameters such as TMR and J_C, we compute the coefficient of variability $CV = \frac{standard\ deviation}{mean} * 100$. We find that values of CV are less than 20% for 35 nm sized devices (electrical sizes 32.5 – 37.5) as shown below. Further reduction of variability will be accompanied by the use of CMOS large wafer scale deposition and lithography tools for fabricating these MTJs.

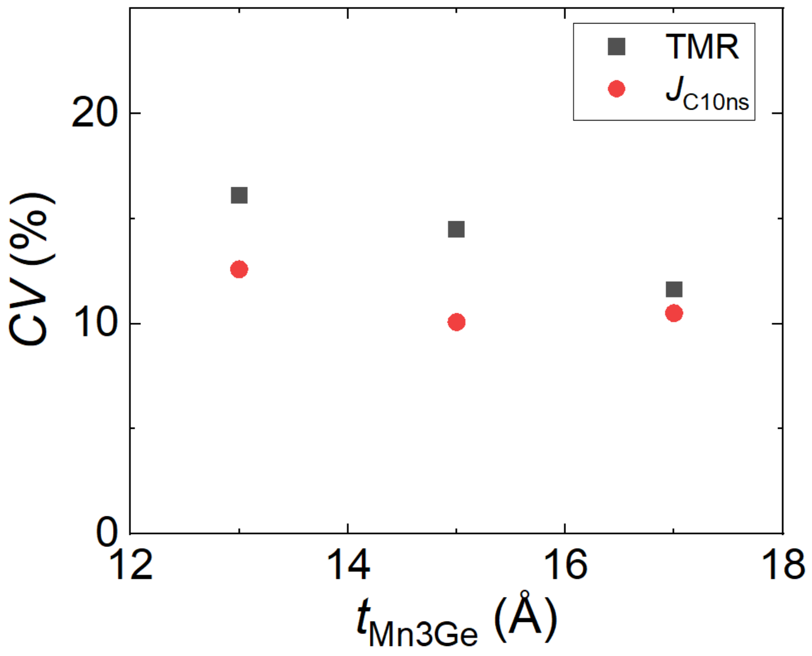

Supplementary Figure 9. Coefficient of variability for TMR and J_C

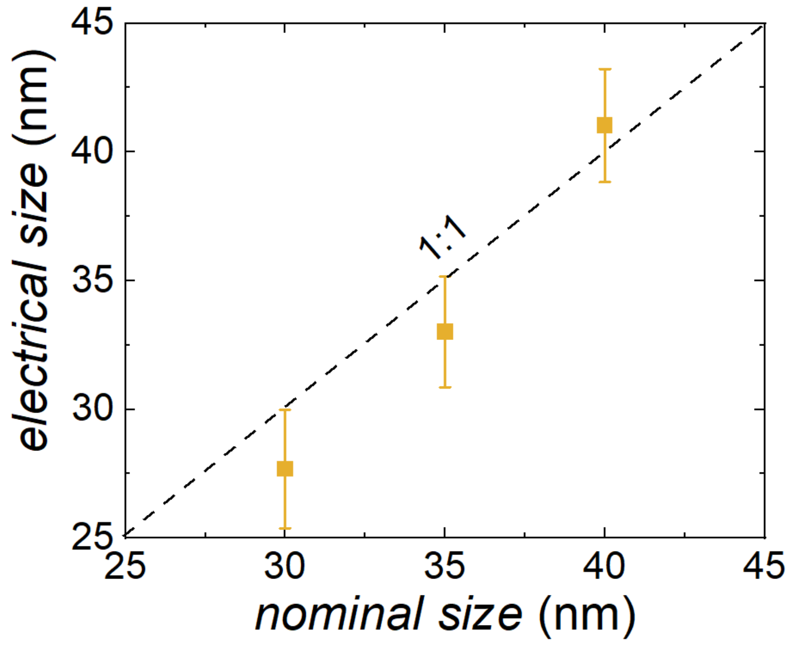

Supplementary Figure 10. Nominal vs electrical size comparison for devices fabricated for the t_{Mn_3Ge} = 17 Å stack. The mean electrical sizes for the nominal sizes 30, 35 and 40 nm (sample sizes of 40 or more in number) are within 10% and thus the effect of windage and possible oxidation of the tunnel barrier is not significant. The error bars correspond to one standard deviation.

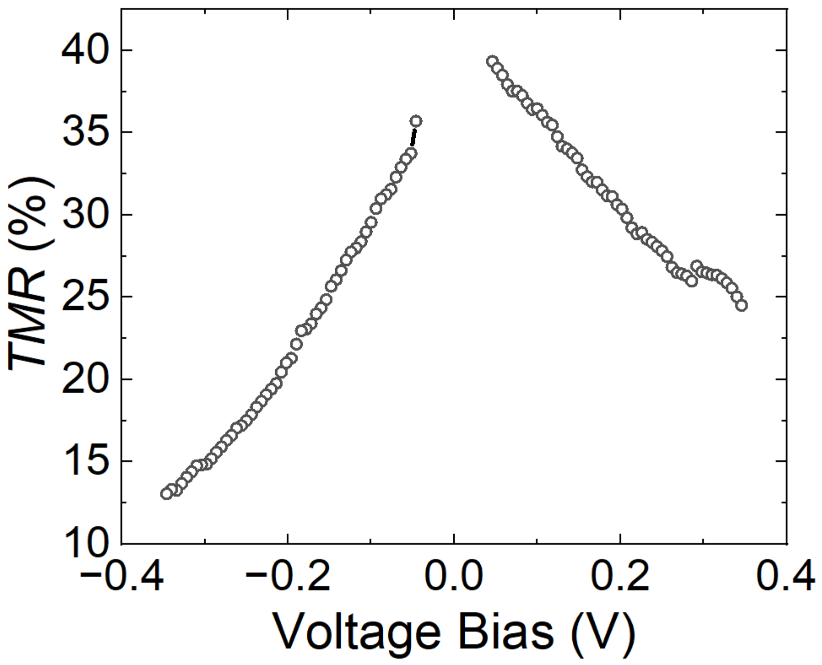

Supplementary Figure 11. Biased dependence of TMR for a representative device from MTJ stack with t_{Mn_3Ge} = 17 Å. The TMR values were derived from the P and AP resistance values at different voltage bias and room temperature condition. The TMR drops sharply with voltage

bias reducing to a value of ~25% for 0.3V and a value of ~15% for -0.3V and thus exhibits an asymmetry with respect to the voltage bias polarity.

Supplementary Note 5: Size dependence of device E_B

We measure the size-dependence of E_B for t_{Mn_3Ge} = 17 Å stack by considering three sizes - 30, 35 and 40 nm - where each of these sizes encompass a set of devices with the electrical size ranges 27.5 – 32.5, 32.5 – 37.5 and 37.5 – 42.5 respectively. This binning is done to aggregate statistics. For size groups 35 and 40 nm, we see a flat dependence of E_B and start to see a slight decrease for 30 nm. Typically, a decrease in E_B with size reflects the macrospin reversal of the magnetic free layer as the energy barrier decreases with the area of the junction. However, when the junction diameter is bigger than the exchange length of the magnetic layer, sub-volume activation can lead to a saturation of E_B with increasing size. Measurements on junctions smaller than 30 nm are needed to confirm the crossover from macospin to sub-volume reversal.

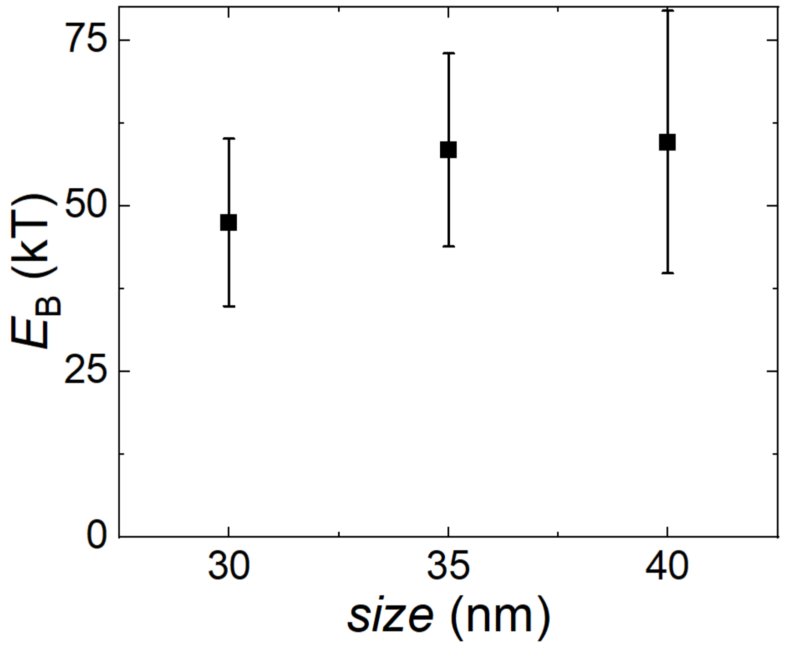

Supplementary Figure 12. Mean E_B for three different sizes (sample sizes of 20 or higher). Error bars in the figure correspond to one standard deviation.

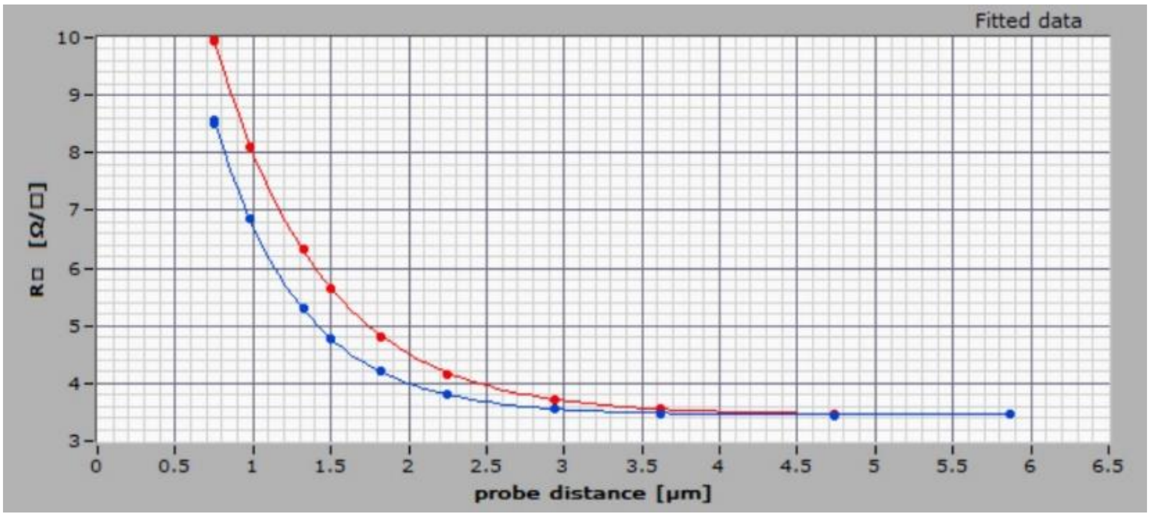

Supplementary Figure 13. CIPT results as obtained from the SmartTip CIPT measurement tool. Fitting parameters are: sheet resistances for top layer, Rt: 17.860 Ω/□, and bottom layer, Rb: 4.282 Ω/□. Resistance Area product for the Parallel state, RA^P: 8.497 Ω μm^2, and anti-parallel state, RA^{AP}:12.801 Ω μm^2. TMR: 50.655 %. Characteristic length scale, λ: 0.62 μm, where $\lambda = \sqrt{\frac{RA}{R_t+R_b}}$ and coefficient of determination of the fit R^2: 1.

References

[1] J. Jeong, Y. Ferrante, S. V. Faleev, M. G. Samant, C. Felser, and S. S. P. Parkin, Nature Comm. 7, 10276 (2016)

[2] G. Kresse and J. Furthmüller, Phys. Rev. B **54**, 11169 (1996).

[3] P. E. Blochl, Phys. Rev. B **50**, 17953 (1994).

[4] G. Kresse and D. Joubert, Phys. Rev. B **59**, 1758 (1999).

[5] J. P. Perdew, K. Burke, and M. Ernzerhof, Phys. Rev. Lett. **77**, 3865 (1996).

[6] S. V. Faleev, Y. Ferrante, J. Jeong, M. G. Samant, B. Jones, and S. S. P. Parkin, Phys. Rev. Applied 7, 034022 (2017).

[7] M. Methfessel *et al.*, in *Lecture Notes in Physics*, edited by H. Dreysse (Springer-Verlag, Berlin, 2000), Vol. 535.

[8] U. von Barth and L. Hedin, J. Phys. C **5**, 1629 (1972).

[9] S. V. Faleev, M. van Schilfgaarde, and T. Kotani, Phys. Rev. Lett. **93**, 126406 (2004).

[10] M. van Schilfgaarde, T. Kotani, and S. V. Faleev, Phys. Rev. Lett. **96**, 226402 (2006).

[11] T. Kotani, M. van Schilfgaarde, and S. V. Faleev, Phys. Rev. B**76**, 165106 (2007).

[12] Turek, I., Drchal, V., Kudrnovsky, J., Sob, M. & Weinberger, P. *Electronic structure of disordered alloys, surfaces and interfaces* (Kluwer, 1997).

[13] Schilfgaarde, M. v. & Lambrecht, W. R. L. in *Tight-binding approach to computational materials science* vol. 491 (eds Colombo, L., Gonis, A. & Turchi, P.) 137 (MRS, 1998).

[14] Y. Miura, and M. Shirai, IEEE Trans. Magn. 50, 1400504 (2014).

[15] D. Wortmann, G. Bihlmayer and S. Blugel, J. Phys. Condens. Matter 16, S5819–S5822 (2004).
